# Thermoresponsive Gel for Sustained Nanoliposome-Mediated Nose-to-Brain Drug Delivery

**DOI:** 10.3390/ijms27219759

**Published:** 2026-10-30

**Authors:** Ljubica Mihailova, Dushko Shalabalija, Marija Glavas Dodov, Milena Mishonova, Lea Koceva, Neli Raikova, Mitko Mladenov, Hristo Gagov, Jana Tchekalarova, Polina Petkova-Kirova, Iliyana Sazdova

**Affiliations:** 1 Institute of Pharmaceutical Technology, Faculty of Pharmacy, Ss. Cyril and Methodius University in Skopje, Majka Tereza 47, 1000 Skopje, North Macedonia; lj.mihailova@ff.ukim.edu.mk (L.M.); d.shalabalija@ff.ukim.edu.mk (D.S.); magl@ff.ukim.edu.mk (M.G.D.); 2 Department of Animal and Human Physiology, Faculty of Biology, Sofia University “St. Kliment Ohridski”, 8 Dragan Tsankov Blvd., 1164 Sofia, Bulgaria; mmishonova@biofac.uni-sofia.bg (M.M.); lealk@uni-sofia.bg (L.K.); neliraikova@biofac.uni-sofia.bg (N.R.); hgagov@uni-sofia.bg (H.G.); 3 Institute of Biology, Faculty of Natural Sciences and Mathematics, Ss. Cyril and Methodius University in Skopje, 1000 Skopje, North Macedonia; mitkom@pmf.ukim.mk; 4 Institute of Neurobiology, Bulgarian Academy of Sciences, Acad. G. Bonchev Str., Bl. 23, 1113 Sofia, Bulgaria; janetchekalarova@gmail.com

**Keywords:** thermoresponsive gel, near-infrared fluorescence, olfactory bulb, nasal mucosa, trigeminal nerve, brainstem, nanoliposomes, indocyanine green, nose-to-brain, neurodegenerative diseases

## Abstract

Nose-to-brain drug delivery represents a promising non-invasive strategy for improving CNS targeting. However, conventional liquid formulations are limited by rapid mucociliary clearance and short mucosal residence time. This study developed a thermoresponsive in situ gel incorporating indocyanine green (ICG)-loaded nanoliposomes and evaluated its potential to enhance and modulate the ICG-associated brain distribution after intranasal administration of the formulation. ICG-loaded nanoliposomes were prepared using a modified thin-film hydration method and incorporated into a poloxamer-based gel containing hyaluronic acid and Carbomer 940 as mucoadhesive components. The nanoliposomes exhibited a mean particle size of 124 ± 3.25 nm, a unimodal size distribution, and a high ICG encapsulation efficiency of 93.33%. Rheological analysis confirmed a temperature-triggered sol-to-gel transition at the physiological nasal temperature (~32 °C), shear-thinning behavior, and rapid structural recovery suitable for intranasal application. Ex vivo fluorescence imaging in rats demonstrated distinct biodistribution profiles for the ICG-associated signal delivered via the liquid nanoliposomes and gel-incorporated nanoliposomes. Although the liquid liposomes showed faster early signal at 4 h, the gel formulation produced stronger and more sustained brain fluorescence at later time points. At 8 h and 24 h, whole-brain fluorescence was approximately twofold higher with the gel formulation than with the liquid liposomes formulation. These findings indicate that this thermoresponsive gel enhances nasal retention and may modulate the delivery profile of the nose-to-brain transport, supporting its potential as a platform for CNS-targeted delivery and the development of more effective therapies for neurodegenerative diseases.

## 1. Introduction

The targeted delivery of therapeutic agents to the central nervous system (CNS) remains one of the most formidable challenges in contemporary neuropharmacology. The blood–brain barrier (BBB), characterized by brain capillary endothelial cells with tight junctions, efflux transporters, and a lack of fenestrations, severely restricts the entry of many small-molecule drugs and most large or hydrophilic therapeutic agents into the brain [1,2]. Consequently, conventional systemic administration approaches often require high doses to achieve therapeutic thresholds, escalating peripheral off-target toxicities and curtailing clinical efficacy [3].

To circumvent the BBB, the intranasal route has emerged as a promising, non-invasive strategy for direct nose-to-brain macromolecular delivery [4]. This approach exploits the unique anatomical architecture of the olfactory and trigeminal neuroepithelia, which provide a direct cellular bridge between the external nasal cavity and the CNS [5,6]. By utilizing these neural pathways, intranasally administered substances can access the brain via intracellular axonal transport or extracellular bulk flow through perineural spaces, effectively bypassing the systemic circulation and avoiding hepatic first-pass metabolism [7].

Anatomically, the olfactory pathway occupies the superior turbinate, where olfactory receptor neurons project through the cribriform plate into the olfactory bulb, establishing a direct connection with forebrain structures [8]. Nevertheless, the nasal environment also presents several physiological barriers that significantly influence drug transport, including mucus composition, epithelial integrity, enzymatic activity, immune surveillance, and mucociliary clearance. Therefore, successful nose-to-brain delivery requires careful optimization of both the formulation and its interaction with the nasal microenvironment [9,10,11,12]. Concurrently, the trigeminal nerve extensively innervates both the respiratory and olfactory mucosa, entering the CNS via the pons, providing therapeutic access to caudal brain structures and the brainstem [13]. The relative contribution of these pathways is largely governed by the physicochemical properties of the formulations and their spatial deposition within the nasal cavity [14].

Despite these anatomical advantages, the clinical translation of conventional intranasal liquid formulations is severely constrained by the innate clearance mechanisms of the nasal mucosa [15]. Continuous mucus secretion and coordinated ciliary beating drive a high mucociliary clearance rate that rapidly expels exogenous formulations, drastically shortening the window available for mucosal absorption [16]. Furthermore, the complex viscoelastic mucus hydrogel acts as a physical and enzymatic barrier, trapping or degrading nanocarriers before they can access neuronal pathways [17]. Therefore, overcoming these barriers requires the engineering of advanced biomaterials that simultaneously prolong mucosal retention and enhance structural permeability [18].

These physiological limitations have stimulated the development of nanoscale carrier systems capable of protecting therapeutic agents and enhancing their interaction with the nasal epithelium. In recent years, the integration of lipid-based nanocarriers within stimuli-responsive supramolecular hydrogels has emerged as a sophisticated paradigm in nanomedicine [19]. In this regard, liposomes offer exceptional biomimetic biocompatibility, low immunogenicity, and the capacity to encapsulate structurally diverse hydrophilic and lipophilic cargos within their aqueous core and lipid bilayer, respectively, protecting them from localized enzymatic degradation. However, when deployed as an aqueous suspension, liposomal vectors remain highly susceptible to rapid mucociliary clearance [20].

Therefore, embedding these nanostructures within a thermosensitive in situ gelling polymeric matrix directly addresses this limitation. These smart biomaterials undergo a concentration-dependent, thermally induced reversible sol-to-gel transition, existing as a low-viscosity liquid at ambient temperature to facilitate precise atomization, while rapidly transitioning into a viscoelastic hydrogel network upon exposure to physiological nasal temperatures [21,22]. This phase transition is typically driven by amphiphilic block copolymers, such as poloxamers, which undergo self-assembly into spherical micelles as the temperature rises; subsequent micellar packing and entanglement form a rigid three-dimensional network [23]. The resultant polymeric matrix structurally anchors the liposomes to the nasal epithelium, significantly extending mucosal residence time, suppressing mucociliary clearance, and ensuring robust localized retention of the encapsulated agent within a 24-h window [24].

Several nose-to-brain hydrogel systems based on thermoresponsive and/or mucoadhesive polymers have been investigated to increase nasal residence time and reduce mucociliary clearance. For example, thermoreversible intranasal gels based on Poloxamer 407 and Carbopol have been developed to provide temperature-dependent gelation together with mucoadhesive properties, while liposome-loaded thermosensitive nasal hydrogels have also been investigated for brain delivery [21,25]. More recently, Lutrol 127 (Poloxamer 407)-based gels incorporating Carbomer and hyaluronic acid have been evaluated for intranasal brain delivery [26]. However, comparative evaluation of such systems against conventional nanocarriers dispersions, particularly with respect to their regional brain distribution following intranasal administration, remains limited. In the present study, Poloxamer 407 and Poloxamer 188 were combined with low-molecular-weight hyaluronic acid and Carbomer 940 to provide a thermoresponsive and mucoadhesive matrix for incorporation of liposomal nanocarriers. The developed formulation was subsequently evaluated in vivo and directly compared with the corresponding liposomal dispersion.

Such comparative evaluation is important because the incorporation of a nanocarrier into a hydrogel matrix may alter its nasal retention and subsequent distribution within the CNS. Precise mapping of the biodistribution of delivered agents across discrete brain regions can therefore provide valuable information on formulation performance and the potential contribution of the intranasal route to CNS delivery. Fluorescent imaging enables visualization of the distribution of administered compounds within the nasal cavity, olfactory bulbs, brainstem, other brain regions, and peripheral organs, providing spatial information relevant to the assessment of direct nose-to-brain delivery and systemic redistribution [27]. Such spatial mapping is particularly relevant for evaluating novel nanocarrier-based intranasal platforms intended for CNS-targeted delivery.

To address these gaps, this study introduces a dual-functioning nanostructured platform designed for targeted CNS delivery by embedding liposomes loaded with a hydrophilic marker dye within a thermosensitive hydrogel matrix. Following rigorous in vitro physicochemical and rheological characterization, the experimental framework includes comparative in vivo evaluations and precise spatial mapping of brain distribution in murine models. In this context, a direct, head-to-head comparison between the composite thermosensitive liposomal gel architecture and conventional liposomal dispersions was performed to systematically evaluate the translational utility of the direct nose-to-brain route.

## 2. Results

### 2.1. Characterization of ICG-Loaded Nanoliposomes

The prepared ICG-loaded nanoliposomes showed a mean particle size, expressed as D50, of 124 ± 3.25 nm. Laser diffraction analysis demonstrated a unimodal particle size distribution, with a SPAN value of approximately 1, indicating a relatively narrow and homogeneous size distribution in the nanoliposomal dispersion. Complementary dynamic light scattering (DLS) analysis using a Zetasizer showed a comparable mean particle size of 129.33 ± 1.23 nm, with a polydispersity index (PDI) of 0.28 ± 0.04, further confirming a relatively narrow size distribution. The measured zeta potential was −14.8 ± 1.43 mV, indicating a moderately negative surface charge of the nanoliposomes. These results confirm the successful preparation of nanosized liposomal carriers suitable for further incorporation into the thermoresponsive gel and subsequent in vitro and in vivo evaluation. The ICG-loaded nanoliposomes exhibited high encapsulation efficiency, with 93.33% ± 3.2% (n = 3) of the initially added dye retained within the nanoliposomal formulation. Based on the measured EE, approximately 2.80 mg of ICG was associated with the nanoliposomes. Considering the total mass of the lipid phase, the ICG loading was calculated as 0.82% (*w*/*w*).

### 2.2. Linear Viscoelastic Region Profiling (LVR) and Amplitude Sweeps

Oscillatory amplitude sweeps were conducted at a constant frequency to establish the linear viscoelastic region (LVR) and define the structural sensitivity of the formulations to mechanical deformation.

**Figure 1:**
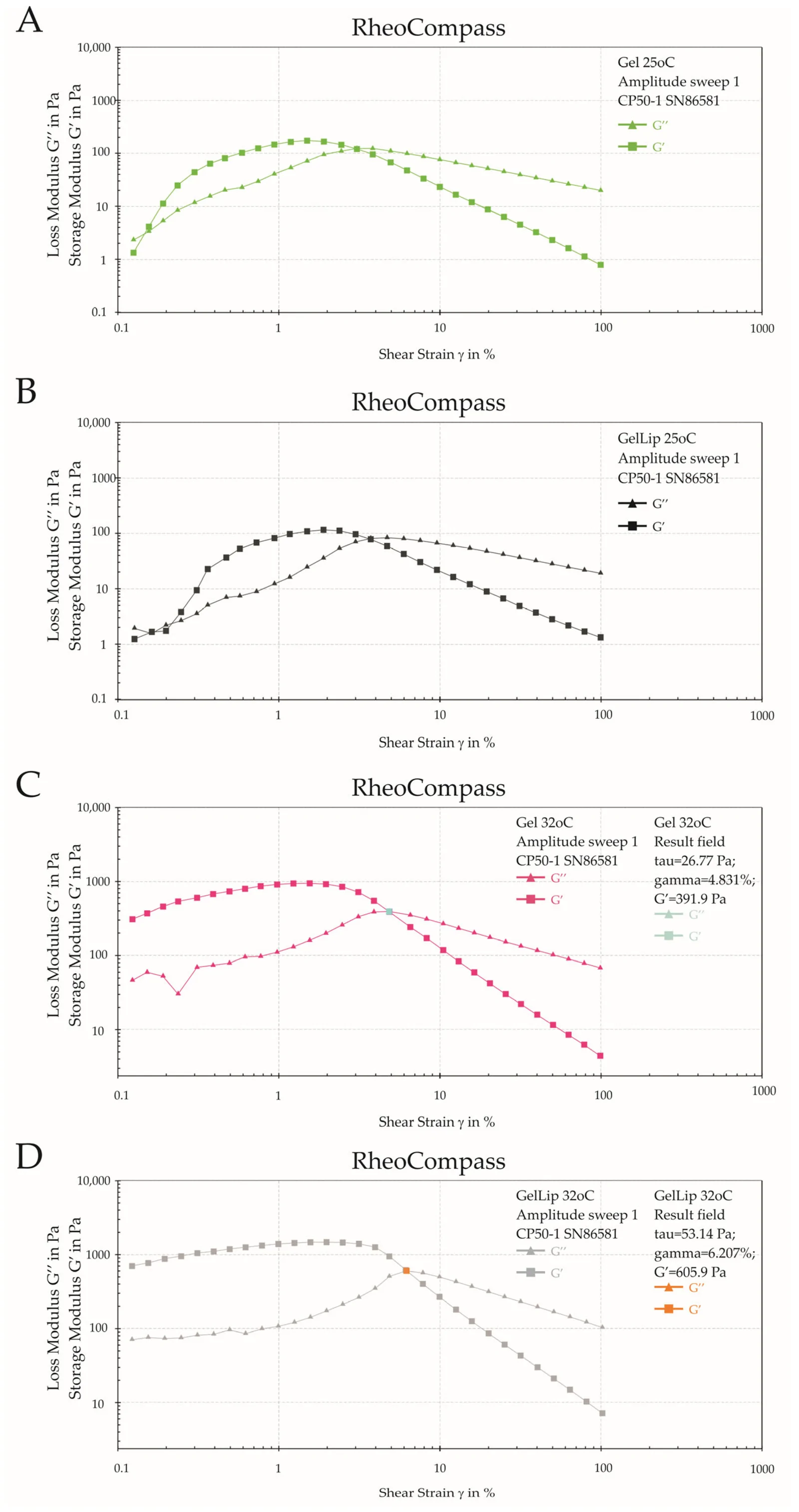
Oscillatory amplitude sweeps of the (**A**) thermoresponsive gel (Gel) at 25 °C; (**B**) thermoresponsive gel loaded with nanoliposomes (GelLip) at 25 °C; (**C**) thermoresponsive gel (Gel) at 32 °C; (**D**) thermoresponsive gel loaded with nanoliposomes (GelLip) at 32 °C as a function of shear strain (γ: 0.01% to 100%).

At ambient temperature (25 °C), the thermoresponsive gel (Gel) demonstrated early partial structural network assembly, with G′ marginally exceeding G″ at a baseline plateau of 4.6–4.7 Pa (tanδ approx. 0.61–0.62; η* approx 860–880 mPa·s) (Figure 1A). Conversely, the composite system (GelLip) exhibited a definitive liquid state. After the resolution of initial motor compliance artifacts, the loss modulus (G″) consistently dominated the storage modulus (G′), maintaining a stable horizontal plateau between 1.78 and 1.83 Pa, whereas G′ remained at a baseline of 0.18–0.40 Pa. This behavior was confirmed by a high loss factor (tanδ) ranging from 4.5 to 9.7 and a low complex viscosity (η*) baseline of 250–280 mPa·s (Figure 1B).

Upon shifting to the physiological nasal temperature (32 °C), both systems underwent a substantial increase of several orders of magnitude in viscoelastic parameters, establishing rigid LVR plateaus. The Gel was characterized by G′ ≫ G″, forming a stable plateau at 254–255 Pa, with G″ remaining significantly lower at 38–39 Pa, yielding a highly elastic loss factor (tanδ) of 0.15 and a complex viscosity (η*) of 40,500–40,700 mPa·s (Figure 1C). For the thermoresponsive gel loaded with nanoliposomes (GelLip), incorporation of the lipid nanocarriers slightly reduced the storage modulus plateau to 191–195 Pa (tanδ approx 0.20; η* approx 31,200–31,800 mPa·s) (Figure 1D). In rheological terms, at 32 °C, both formulations successfully transitioned into structurally stable, highly elastic hydrogel networks that displayed solid-like viscoelastic properties within the LVR up to a critical deformation threshold (γc) of approximately 1.0% before entering the nonlinear viscoelastic region and initiating network collapse.

### 2.3. Thermal Phase Transition Dynamics (Temperature Sweeps)

Continuous temperature-sweep testing monitored the sol-to-gel transition as a function of thermal energy, from ambient temperatures to hyperthermic thresholds (~40 °C).

In the low-temperature sol state (<30 °C), both formulations behaved as low-viscosity fluids dominated by viscous components (G″ > G′). The Gel formulation exhibited an intermediate baseline viscosity before undergoing a sharp, thermally induced phase transition. The rheological gelation temperature (Tgel), defined as the exact thermodynamic crossover point at which G′ = G″, occurred at 31.1 °C (modulus approx 1.8 Pa).

The integration of liposomal nanocarriers altered the self-assembly thermodynamics, shifting the transition threshold downward. The GelLip formulation demonstrated a Tgel of 30.8 °C (modulus approx 0.85 Pa). Above their respective crossover temperatures, both systems displayed a sudden, steep, parallel increase in viscoelastic properties. At 34.0 °C, the Gel reached a G′ of ~31 Pa (G″ approx 9.7 Pa), while the GelLip achieved a comparable G′ of ~33 Pa (G″ approx 11 Pa). At the terminal testing temperature (~39.5 °C), the maximum storage modulus plateaued at 267 Pa for the Gel and 195 Pa for the GelLip.

### 2.4. Steady-State Rotational Flow Curve Analysis

Rotational flow curve analyses at the physiological temperature (32 °C) evaluated the formulation viscosity η over an operational shear rate sweep (γ) from 1 to 1000 s^−1^.

**Figure 2:**
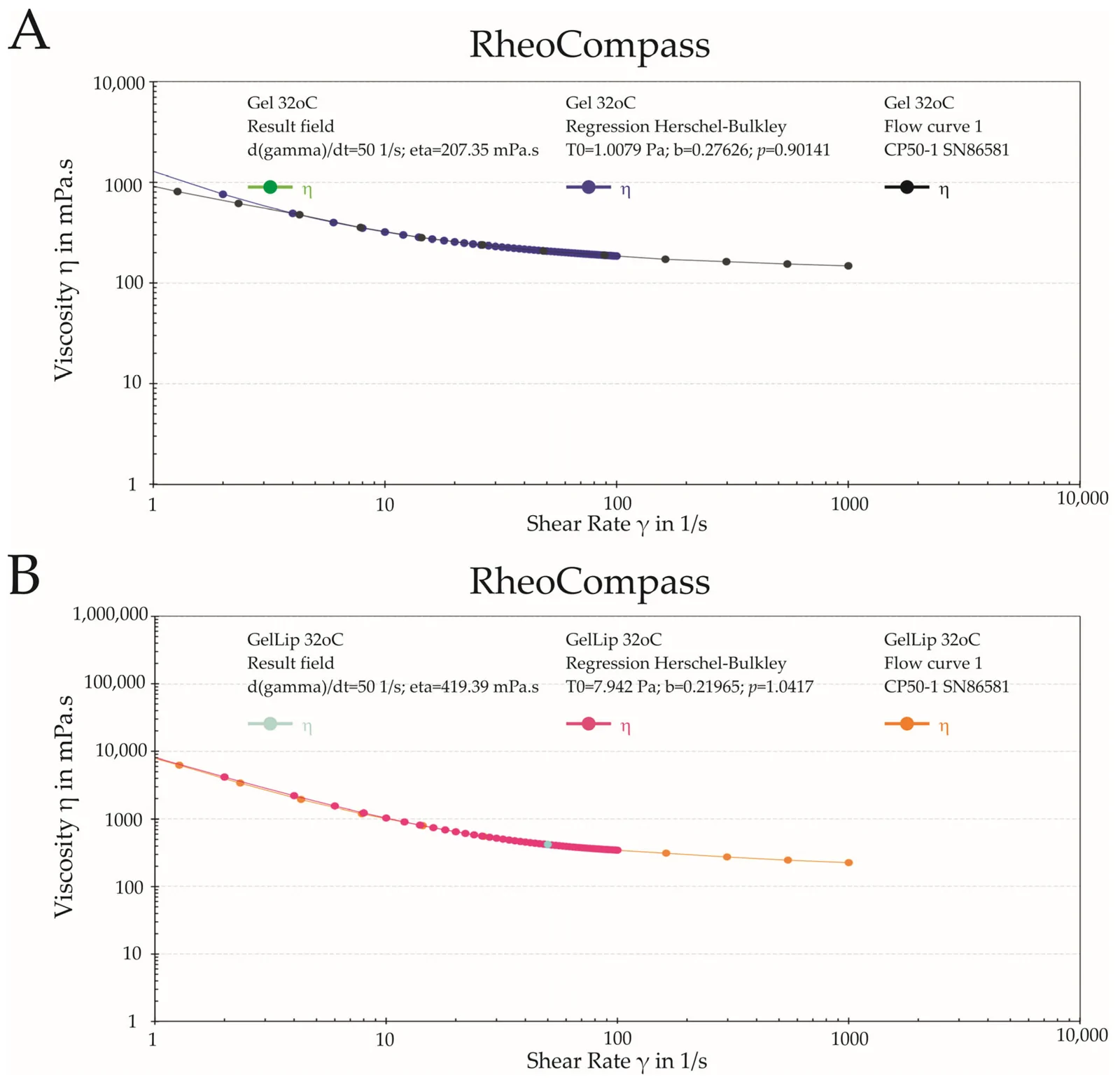
Steady-state rotational flow curves showcasing dynamic viscosity (η) and shear stress (τ) over an operational shear rate sweep γ = 1 to 1000 s^−1^ of (**A**) thermoresponsive gel (Gel) and (**B**) thermoresponsive gel loaded with nanoliposomes (GelLip) at 32 °C.

Both formulations demonstrated non-Newtonian, pseudoplastic (shear-thinning) profiles, characterized by a continuous decline in viscosity across several orders of magnitude as the shear rate increased. However, the magnitude of resistance to flow was markedly altered by the inclusion of lipid vesicles. At the initial low-shear point γ = 1 s^−1^, the GelLip displayed a highly structured baseline viscosity of approximately 8000 mPa·s (Figure 2B), which was roughly eightfold higher than that of the Gel (~1000 mPa·s) (Figure 2A). At an intermediate operational reference shear rate γ = 50 s^−1^, the GelLip maintained a significantly higher viscosity (419.39 mPa·s) than the Gel (207.35 mPa·s). At the ultra-high shear boundary (1000 s^−1^) the viscosities of the GelLip and Gel converged to fluid baselines of 220 mPa·s and 140 mPa·s, respectively.

Fitting the experimental flow curves to the mathematical Herschel–Bulkley regression model quantified these structural discrepancies. The Gel exhibited a low yield stress τ0 of 1.0079 Pa and a classic flow index (p) of 0.90141. Conversely, the GelLip demonstrated an eightfold higher yield stress τ0 of 7.942 Pa, combined with a recalculated flow index (K) of 1.0417.

### 2.5. Three-Step Interval Thixotropy Testing (3ITT)

The time-dependent structural breakdown and kinetic rebuilding of the formulations were evaluated using a three-step interval thixotropy test (3ITT) at both 25 °C and 32 °C.

**Figure 3:**
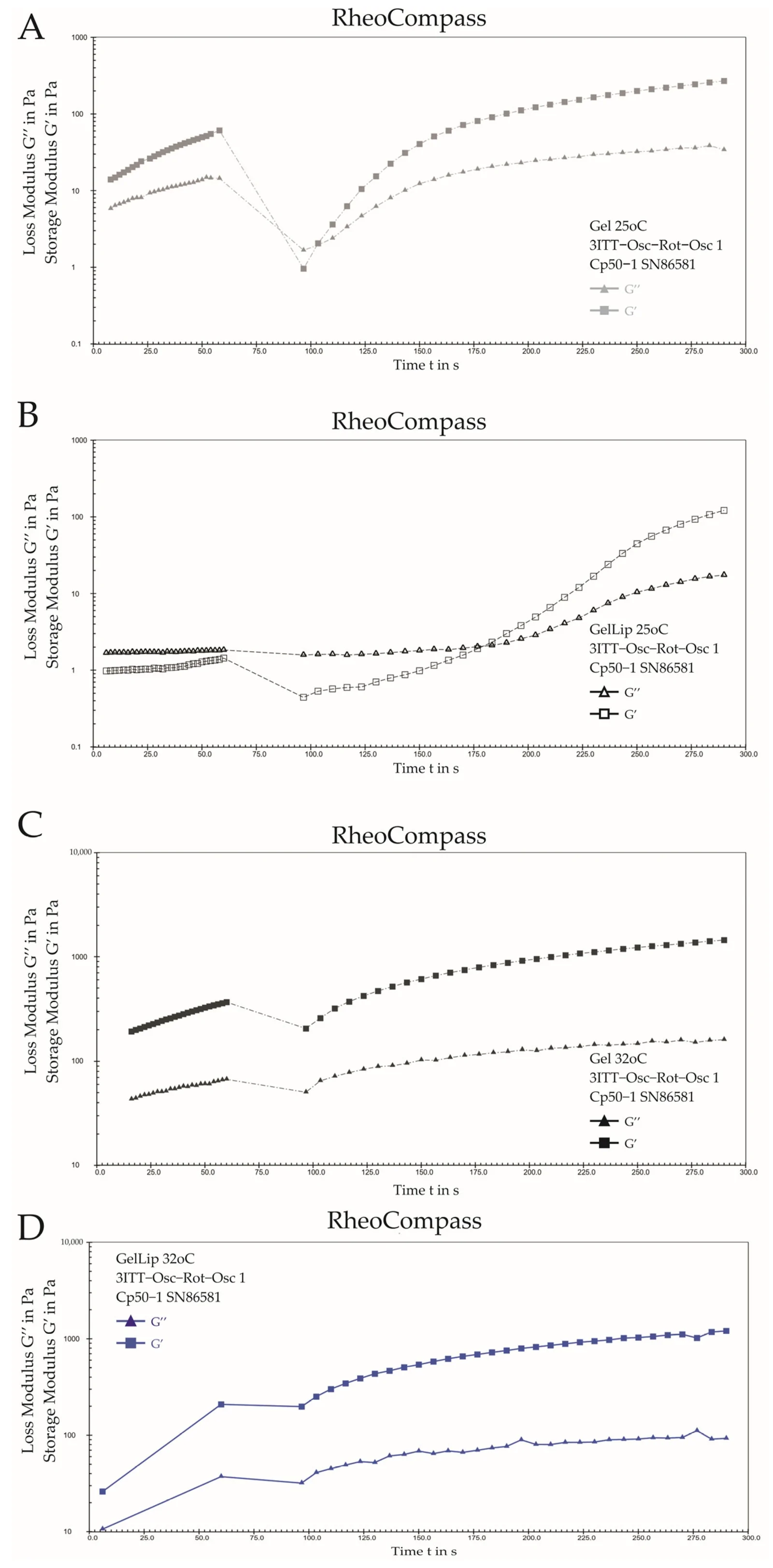
Three-Step Interval Thixotropy Test (3ITT) evaluating time-dependent structural breakdown and kinetic rebuilding profiles of (**A**) thermoresponsive gel at 25 °C (Gel 25oC), (**B**) thermoresponsive gel loaded with nanoliposomes at 25 °C (GelLip 25oC); (**C**) thermoresponsive gel at 32 °C (Gel 32oC); (**D**) thermoresponsive gel loaded with nanoliposomes at 32 °C (GelLip 32oC).

At ambient temperature (25 °C), the Gel showed early partial structuring during Interval 1, dropped to a G′ of 1 Pa under high shear, and recovered more rapidly to a terminal plateau of 280 Pa (Figure 3A). In contrast, the GelLip maintained a fluid baseline during Interval 1 (G″ > G′), dropped to a minimum G′ of 0.45 Pa under high shear in Interval 2, and exhibited a markedly delayed, incomplete structural recovery in Interval 3, reaching a maximum G′ of only 120 Pa after 200 s of rest (Figure 3B).

At the physiological temperature (32 °C), both systems exhibited classic thixotropic properties (Figure 3C,D). During Interval 1, the Gel stabilized at an elastic baseline (G′ = 380 Pa), decreased to an intermediate value of 210 Pa under high shear, and immediately recovered to 600 Pa within 50 s of shear cessation, ultimately plateauing at 1450 Pa. The GelLip stabilized at a baseline of 220 Pa in Interval 1, but underwent a much deeper structural breakdown during Interval 2, with G′ dropping directly to 50 Pa. Upon entering the recovery phase (Interval 3), the GelLip exhibited rapid, highly efficient structural rebuilding kinetics, rapidly returning to the elastic domain (G′ ≫ G″). It reached a structural value of 500 Pa within 50 s and stabilized at a final plateau of 1200 Pa.

### 2.6. Ex Vivo NIR Fluorescence Imaging of ICG in the Rat Nasal Cavity

**Figure 4:**
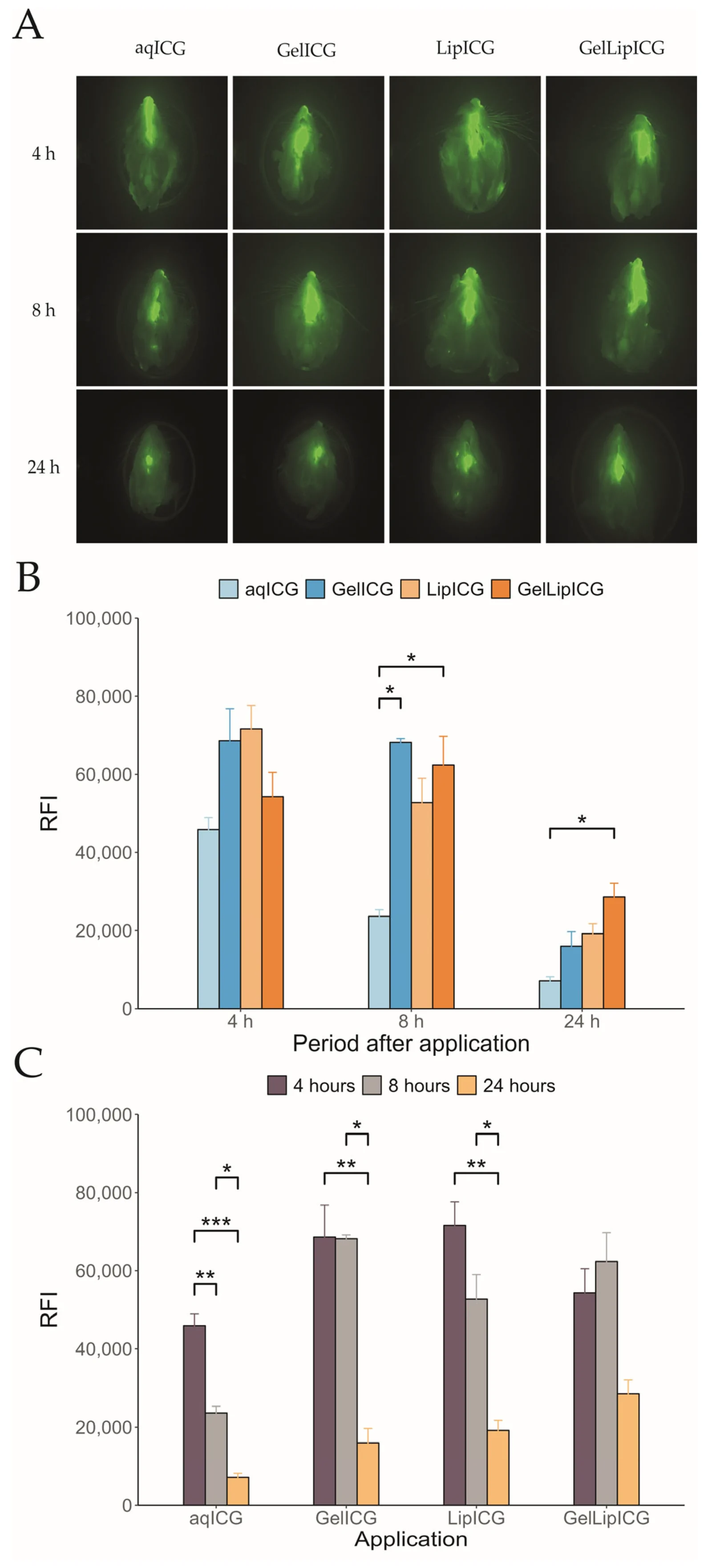
Nasal cavity. (**A**) Ex vivo NIR bioimaging of the nasal cavity at 4 h, 8 h, and 24 h after the application of free ICG solution (aqICG), ICG incorporated into a thermoresponsive gel (GelICG), ICG encapsulated in nanoliposomes (LipICG), or ICG-loaded nanoliposomes incorporated into a thermoresponsive gel (GelLipICG). (**B**) RFI values of ICG fluorescence in the rat nasal cavity obtained at the indicated time points after the administration of aqICG (light blue, n = 6), GelICG (dark blue, n = 6), LipICG (light orange, n = 6), or GelLipICG (dark orange, n = 6). The blue palette represents free ICG, while the orange palette represents ICG encapsulated in nanoliposomes; dark colors indicate gel inclusion, and light colors indicate the free form. (**C**) RFI values of ICG fluorescence in the rat nasal cavity over time for each of the applied formulations of ICG. Data are presented as mean ± standard error of the mean (SEM). * *p* < 0.05, ** *p* < 0.01, *** *p* < 0.001.

Ex vivo near-infrared (NIR) imaging showed a strong ICG fluorescent signal in the rat nasal cavity at 4 h, 8 h, and 24 h following a single intranasal administration (Figure 4). Figure 4A shows representative NIR images of the nasal cavity after ICG application as an aqueous solution (aqICG), ICG incorporated into a thermoresponsive gel (GelICG), ICG encapsulated in nanoliposomes (LipICG), or ICG-loaded nanoliposomes incorporated into a thermoresponsive gel (GelLipICG), with the formulations arranged in columns. The signal intensity was measured as the relative fluorescence intensity (RFI) of the ICG emission at 800 nm. All formulations produced persistent fluorescent signals throughout the observation period. Quantitative analysis of the RFI data showed statistically significant differences between aqICG and the thermoresponsive gel formulations at 8 h after application (Figure 4B). At 24 h after application, a statistically significant difference was found only between aqICG and GelLipICG. The non-gel formulations (aqICG and LipICG) showed a statistically significant time-dependent decrease in the RFI signal in the nasal cavity. Incorporation into a thermoresponsive gel increased the retention of the formulations in the nasal cavity. At 8 h after application, the fluorescent signal was comparable to that observed at 4 h for GelICG (Figure 4C) or even higher than that observed at 4 h for GelLipICG (Figure 4C).

### 2.7. NIR Fluorescence from the Rat Brain

#### 2.7.1. Whole Rat Brain NIR Emission

**Figure 5:**
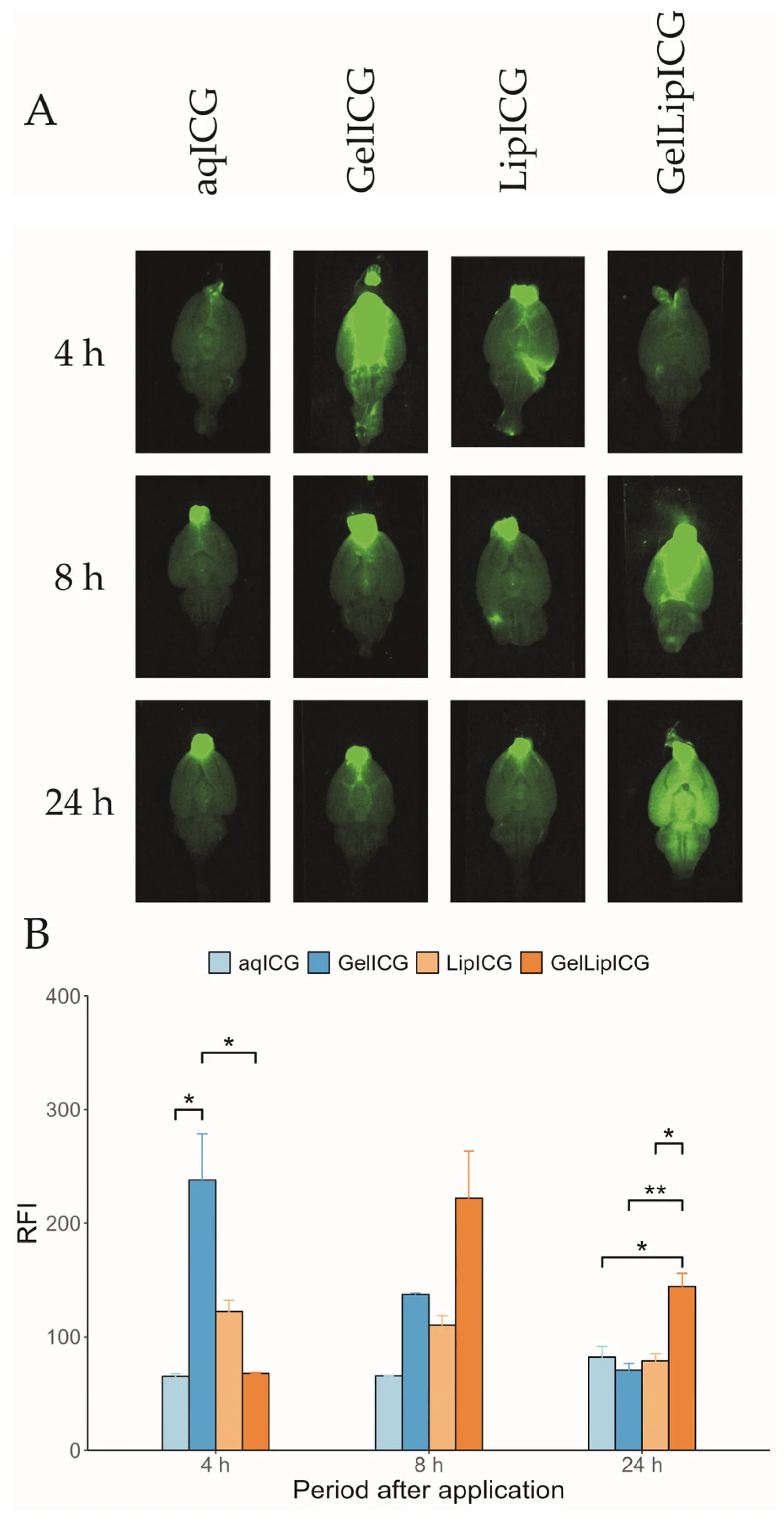
Ex vivo NIR fluorescence images of rat brains. (**A**) Representative NIR fluorescence images after application of ICG (by columns) as an aqueous solution (n = 6), in a thermoresponsive gel (n = 6), in nanoliposomes (n = 6) or as ICG-loaded nanoliposomes in a thermoresponsive gel (n = 6) at 4 h, 8 h, and 24 h (by rows) after intranasal administration. (**B**) RFI values of ICG fluorescence in the rat brain obtained at the corresponding time points after the administration of aqICG (light blue, n = 6), GelICG (dark blue, n = 6), LipICG (light orange, n = 6), or as GelLipICG (dark orange, n = 6). The blue palette represents free ICG, while the orange palette represents ICG encapsulated in nanoliposomes; dark colors indicate gel inclusion, and light colors indicate the free form. Data are presented as mean ± SEM. * *p* < 0.05, ** *p* < 0.01.

Representative NIR fluorescence images of rat brains in ventral view obtained after intranasal administration of ICG are shown in Figure 5A. Fluorescent emission was measured at 4 h, 8 h, and 24 h after a single administration of aqICG, GelICG, LipICG, or GelLipICG by columns. At 4 h post-application, GelICG demonstrated a statistically significant increase in the RFI signal compared with both aqICG and GelLipICG (Figure 5B). At 8 h after application, the fluorescence signals of aqICG and LipICG remained lower, whereas that of GelICG decreased. Conversely, GelLipICG showed a sharp increase. Although no statistically significant difference was found at this specific time point, a notable trend was observed between aqICG and GelLipICG (*p* = 0.0535). At 24 h, GelLipICG demonstrated greater long-term retention, maintaining the highest signal. This retention was significantly higher than that of the other three formulations, all of which showed a decline in fluorescence. The data demonstrate that incorporating ICG into a thermoresponsive gel provides rapid, strong initial retention in the brain at 4 h; encapsulating ICG in liposomes within the gel provides prolonged fluorescence, delaying the peak to 8 h and maintaining a significantly prolonged presence in the brain at 24 h.

#### 2.7.2. NIR Emission from Particular Rat Brain Areas

**Figure 6:**
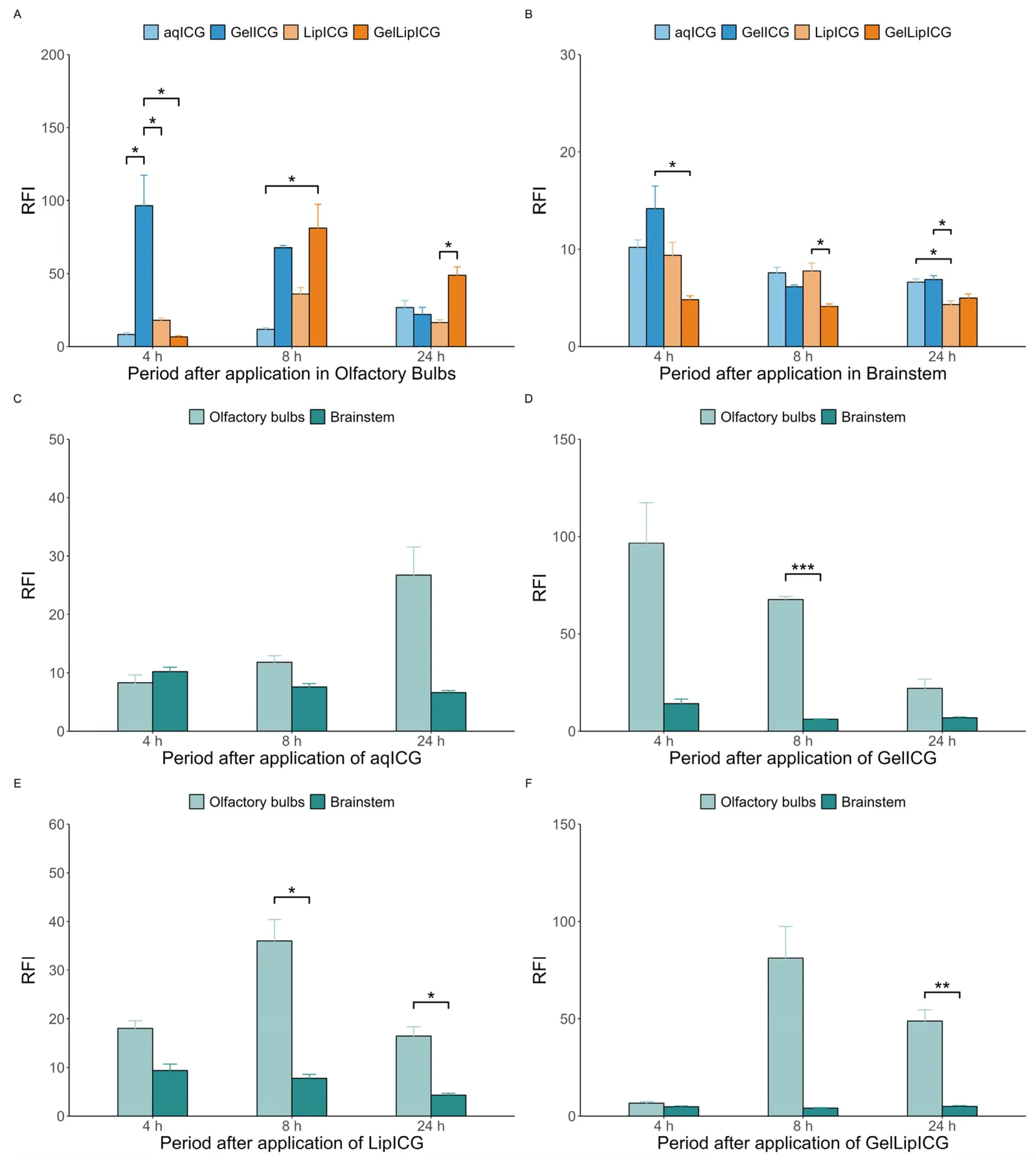
Regional brain distribution of intranasally administered ICG. Relative fluorescence intensity (RFI) of ICG measured in the olfactory bulbs (**A**) and the brainstem (**B**) at 4 h, 8 h, and 24 h after intranasal administration of aqICG (light blue, n = 6), GelICG (dark blue, n = 6), LipICG (light orange, n = 6), or GelLipICG (dark orange, n = 6). The blue palette represents free ICG, while the orange palette represents ICG encapsulated in nanoliposomes; dark colors indicate gel inclusion, and light colors indicate the free form. Relative fluorescence intensity (RFI) of ICG measured after application of aqICG (**C**), GelICG (**D**), LipICG (**E**), or GelLipICG (**F**) in the olfactory bulbs (light teal, n = 6) and in the brainstem (medium teal, n = 6). Statistical comparisons in panels (**A**,**B**) are between the four formulations at each time point, while in panels (**C**–**F**), comparisons are between brain structures for each applied formulation. Data are presented as mean ± SEM. * *p* < 0.05, ** *p* < 0.01, *** *p* < 0.001.

NIR fluorescence originating from the olfactory bulbs (OBs) and brainstem was quantified at three time points (4 h, 8 h, and 24 h) after intranasal administration of aqICG, GelICG, LipICG, or GelLipICG (Figure 6A and B, respectively). At 4 h, GelICG showed the highest fluorescence signal among the tested formulations both in the OBs (Figure 6A) and in the brainstem (Figure 6B). The differences were statistically significant compared with all other formulations in the OBs but only with GelLipICG in the brainstem. At later time points (8 h and 24 h), the strongest fluorescent signal in the OBs was observed for GelLipICG, with statistically significant differences compared with aqICG at 8 h and with LipICG at 24 h. In the brainstem, encapsulation of ICG in liposomes corresponded to a modified signal profile, potentially reflecting a slower transport mechanism associated with the trigeminal pathway. This resulted in significantly lower RFI values than those observed with free ICG, regardless of whether the free ICG was administered as aqICG or GelICG. The higher OB signal suggests a greater contribution of the olfactory pathway to the distribution of all tested ICG formulations compared with the fluorescence accumulation observed in the brainstem (Figure 6C–F). Statistically significant differences were found at 8 h for GelICG and LipICG, and at 24 h for LipICG and GelLipICG.

#### 2.7.3. NIR Emission from Residual Brain Structures

**Figure 7:**
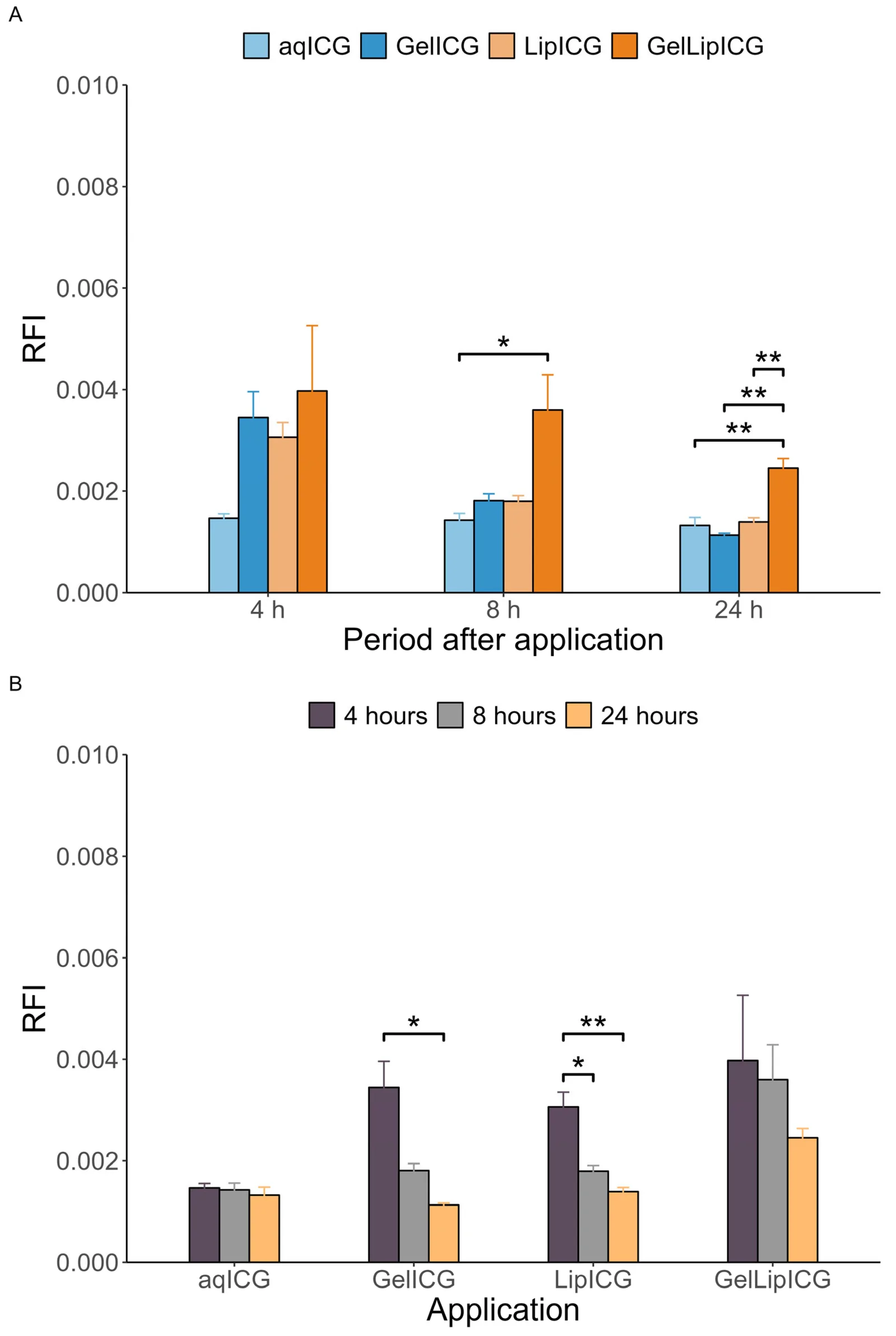
RFI values corresponding to residual fluorescence of the whole brain, excluding olfactory bulbs and brainstem signals, (**A**) following intranasal administration of an aqICG (light blue, n = 6), GelICG (dark blue, n = 6), LipICG (light orange, n = 6) or GelLipICG (dark orange, n = 6). The blue palette represents free ICG, while the orange palette represents ICG encapsulated in nanoliposomes; dark colors indicate gel inclusion, and light colors indicate the free form, presented as time after application. Statistical analyses are between the treatment groups at the respective time intervals. (**B**) Time-dependent changes in the residual signal for each formulation at 4 h (purple, n = 6), 8 h (grey, n = 6), and 24 h (gold, n = 6) after application, presented by application. Statistical analyses are between time intervals within each treatment group. Data are presented as mean ± SEM (n = 6). * *p* < 0.05; ** *p* < 0.01.

To evaluate the distribution of ICG within brain regions other than the olfactory bulbs and brainstem, the signal of the OBs and brainstem was subtracted from the corresponding whole-brain fluorescence signals, and the result was divided by the residual area. The resulting RFI signals were analyzed at 4 h, 8 h, and 24 h after intranasal administration of all tested ICG formulations (Figure 7). Quantitative analysis demonstrated that fluorescence intensities in brain regions beyond the OBs and brainstem were significantly higher in animals treated with GelLipICG than in those treated with aqICG at 8 h post-administration and then in those treated with all other formulations at 24 h after treatment (Figure 7A). This difference likely reflects more efficient overall brain delivery with GelLipICG from the nasal cavity. Additionally, the residual whole-brain signal decreased markedly over time in the groups treated with GelICG and LipICG, with statistically significant differences between time points in each group (Figure 7B). In the group treated with aqICG, the fluorescent signal was the weakest but remained nearly unchanged throughout the observation period. In the group treated with GelLipICG, the fluorescent signal was the strongest and decreased only slightly over time, with the decrease not being statistically significant. Encapsulation of ICG-loaded liposomes within a thermoresponsive gel was associated with enhanced and prolonged brain fluorescence, consistent with increased nasal retention and improved nose-to-brain delivery beyond the primary sites of entry—the olfactory bulbs (OBs) and brainstem.

### 2.8. ICG Biodistribution in Peripheral Organs

**Figure 8:**
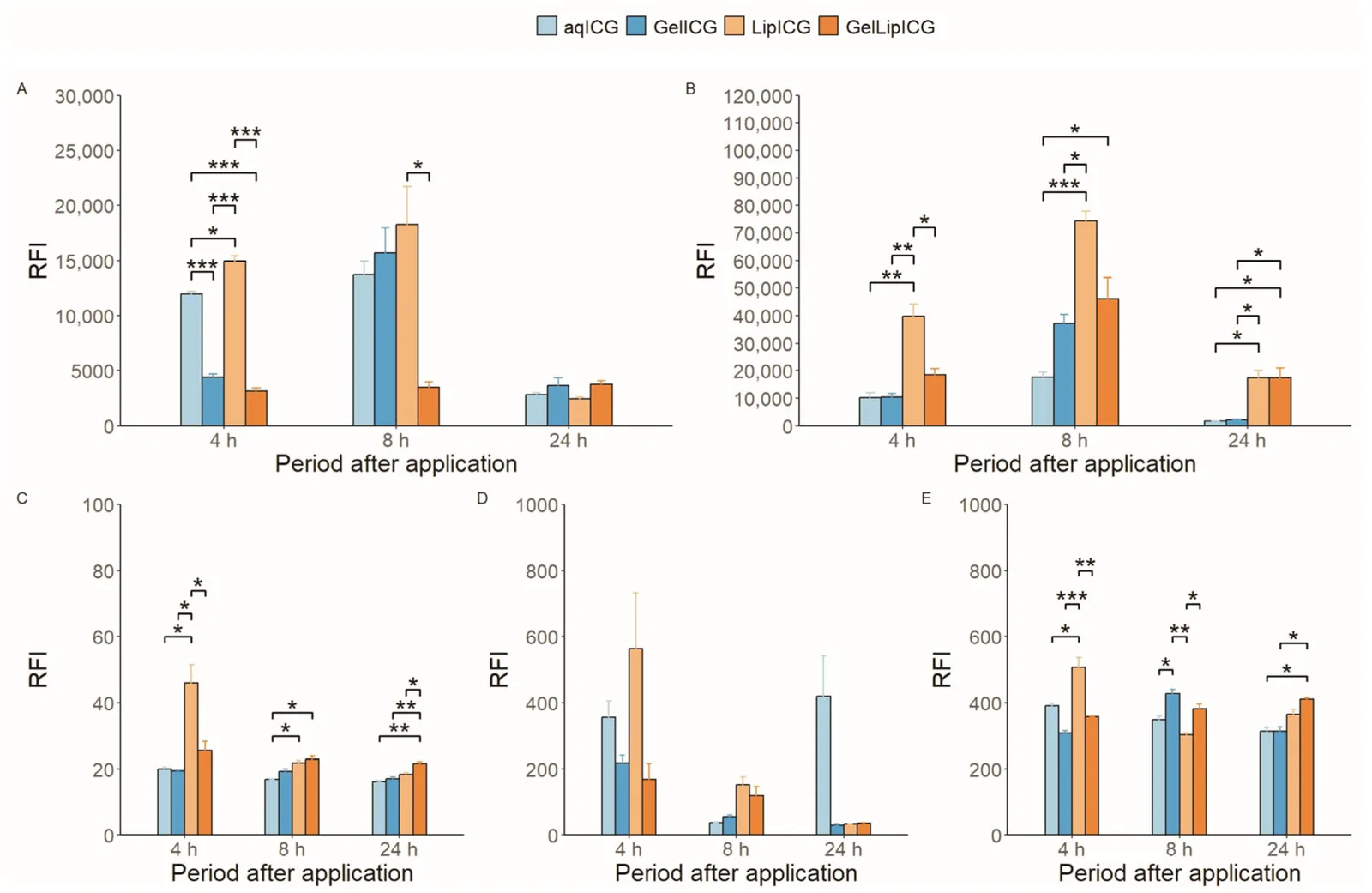
Internal organ biodistribution of intranasally administered ICG. RFI values of ICG fluorescence measured in the liver (**A**), gastrointestinal tract (**B**), heart (**C**), lungs (**D**), and kidney (**E**) at 4 h, 8 h, and 24 h after intranasal administration of aqICG (light blue, n = 6), GelICG (dark blue, n = 6), LipICG (light orange, n = 6), or GelLipICG (dark orange, n = 6). The blue palette represents free ICG, while the orange palette represents ICG encapsulated in nanoliposomes; dark colors indicate gel inclusion, and light colors indicate the free form. Data are presented as mean ± SEM (n = 6). * *p* < 0.05; ** *p* < 0.01, *** *p* < 0.001.

NIR fluorescence was also measured in several peripheral organs following intranasal administration of aqICG, GelICG, LipICG, or GelLipICG (Figure 8). Quantitative analysis demonstrated significantly higher RFI values in the liver of animals treated with formulations lacking the thermoresponsive gel matrix than in those treated with the gel-incorporated formulations during the first 4 h after administration (Figure 8A). At the same time point, LipICG showed a statistically significantly stronger signal compared to all the other formulations. These data indicate that the thermoresponsive gel possibly delays the clearance of ICG from the body, as evidenced also by the much higher RFI values of the GelICG group at 8 h compared to the RFI values of the same group at 4 h. At 8 h the highest RFI value was again for the LipICG group, showing, however, a significant difference only with the GelLipICG group. A significant decrease in the signal from all formulations was observed only at 24 h after application.

Similar results, although shifted in time, were observed in the gastrointestinal tract (Figure 8B), where ICG enters and is excreted in the bile. The LipICG group was again the group showing the highest RFI values, significantly different from most of the other groups both at 4 and 8 h.

In the heart, again, significantly higher RFI values were detected at 4 h in the LipICG group compared to the other groups; however, at the subsequent time points, the values were comparable across all groups (Figure 8C). At 8 h and 24 h, the highest values were detected in the GelLipICG group, but they were several times lower than those observed in the liver and the gastrointestinal tract. In the lungs, no significant differences were observed among the formulation-treated groups over time (Figure 8D).

In the kidneys, the strongest signal was observed in the LipICG group at 4 h after administration (Figure 8E), with significantly higher values than those of all other tested formulations, but at 8 h, the signal significantly decreased compared with both gel-incorporated groups. At 24 h post-application, the GelLipICG group maintained the highest RFI values.

Collectively, these findings indicate that encapsulation of ICG within nanoliposomes accelerates clearance, whereas the depot effect of encapsulation, gel incorporation, or both prolonged the presence of ICG in the bloodstream.

## 3. Discussion

This study presents evidence that:

(1) The developed thermoresponsive in situ gel loaded with nanoliposomes represents a physicochemical and biopharmaceutical advanced intranasal platform, integrating the properties of ICG-loaded nanoliposomes with the shear-dependent flow behavior, rapid in situ gelation, and potential for prolonged nasal residence provided by the thermoresponsive gel matrix. The combination of these features may support the development of a nanoliposome-based intranasal system for nose-to-brain drug delivery.
(2) Intranasal administration of ICG-loaded nanoliposomes incorporated into a thermoresponsive gel produces a stable fluorescent signal from the rat nasal cavity over a period of 24 h. In all other studied groups, the signal declines significantly by 24 h.
(3) Whole-brain NIR emission in rats following intranasal administration of ICG-loaded nanoliposomes incorporated into a thermoresponsive gel at 24 h is strongest and significantly higher compared to all the other groups. The same applies at 24 h for residual brain structures (other than the olfactory bulbs and brainstem). These data suggest better distribution and sustained brain retention with the thermoresponsive gel delivery platform.
(4) The presence of the thermoresponsive gel enhances rapid nose-to-brain delivery of an aqueous solution of ICG and potentially of other small molecules.
(5) ICG-loaded nanoliposomes, but not when delivered with the thermoresponsive gel, in the first hours after intranasal application (4 h and 8 h), result in retention of ICG in the liver and the gastrointestinal tract.
(6) Intranasal administration of nanoliposomes incorporated into a thermoresponsive poloxamer-based gel represents a promising platform for nose-to-brain drug delivery, particularly for potential therapeutic applications where prolonged drug exposure and widespread brain distribution are desirable, such as the treatment of neurodegenerative diseases.

Among the physicochemical characteristics of a nanoliposomal carrier, particle size represents the first critical determinant of whether the system is suitable for intranasal nose-to-brain delivery. Thus, before evaluating the effect of the thermoresponsive gel on nasal retention and brain biodistribution, it was essential to first confirm that the LipICG possessed appropriate nanoscale dimensions and a reproducible particle size distribution. The prepared LipICG exhibited a mean particle size of approximately 124 nm, which falls within the size range generally considered favorable for nasal mucosal interaction, epithelial uptake, and potential transport to the CNS. Several recent reviews indicate that nanocarriers smaller than 200 nm are commonly regarded as suitable for nose-to-brain delivery, as smaller particles may more effectively interact with the nasal epithelium and access transport routes associated with the olfactory and trigeminal regions [28].

The observed particle size is particularly important because intranasal brain delivery depends not only on retention within the nasal cavity but also on the ability of the carrier system to interact with biological barriers and follow direct or indirect transport pathways. Lipid-based nanocarriers, including liposomes, have been widely investigated for intranasal CNS delivery due to their biocompatibility, capacity to encapsulate different types of molecules, and ability to enhance brain deposition compared with conventional drug solutions [29]. In addition, the unimodal distribution is important for reproducible mucosal deposition, cellular uptake, biodistribution behavior, and subsequent interpretation of in vivo fluorescence data. Recent studies further emphasize that a narrow particle size distribution contributes to the predictable absorption and biodistribution of lipid-based nanocarriers administered via the intranasal route [28].

Taken together, the particle size profile of the prepared LipICG supports their suitability as intranasal nanocarriers for nose-to-brain delivery studies. The combination of nanoscale size, unimodal distribution, and liposomal composition provides a rational basis for their use in the thermoresponsive gel system aimed at improving nasal residence, providing prolonged localized delivery, and enhancing brain biodistribution after intranasal administration.

The high encapsulation efficiency obtained in the present study is particularly important considering the physicochemical nature of ICG as a water-soluble/amphiphilic near-infrared fluorescent dye. Efficient incorporation of ICG into liposomal systems is essential not only for providing a strong and reliable fluorescent signal for in vivo biodistribution studies but also for minimizing the presence of free dye, which could diffuse independently of the nanocarrier and confound the interpretation of biodistribution data. Previous studies have shown that liposomal encapsulation can improve the optical and chemical stability of ICG, prolong fluorescence stability, and reduce premature dye leakage compared with free ICG formulations [30]. In this context, the encapsulation efficiency of 93.33% reported in our study indicates that the majority of the fluorescent marker was associated with the nanoliposomal carrier, supporting the suitability of the formulation for tracking nanoliposome-mediated transport and brain biodistribution following intranasal administration. This is especially relevant since the interpretation of brain fluorescence depends on the stability of the LipICG and the assumption that the detected signal primarily reflects carrier-associated transport rather than rapid leakage or redistribution of the free dye.

Regarding the rheological characterization of the prepared thermoresponsive gel, the comparative analysis between ambient (25 °C) and physiological (32 °C) temperatures demonstrates the pronounced thermoresponsive behavior of the engineered system. The increase of several orders of magnitude in the storage modulus (G′) and the complex viscosity (η*) observed in both systems upon heating is driven by the temperature-dependent desolvation of the amphiphilic block copolymer unimers (e.g., poloxamers). At lower temperatures, the entire polymer is soluble in water. As the temperature increases, the hydrophobic poly(propylene oxide) (PPO) segments lose hydration and collapse, forming a spherical micellar core. The poly(ethylene oxide (PEO) chains remain highly hydrated and extended in the aqueous phase, forming a protective “corona” (or shell) around the micelles, which subsequently pack into a rigid, space-filling three-dimensional cubic lattice [31,32,33]. In addition, maintaining a true liquid profile at 25 °C—as seen in the GelLip (tanδ > 4.5; η* < 280 mPa·s)—is important for pharmaceutical stability and clinical usability. It facilitates reliable manufacturing, accurate dosing, and filling into administration devices. Furthermore, a lower viscosity under applied shear may facilitate flow through a nasal delivery device, although actual spray performance depends on both formulation and device-related factors [34,35].

In addition, the underlying supramolecular hydrogel matrix of the thermoresponsive gel is directly influenced by the integration of lipid-based nanocarriers, which modify both the structural and mechanical properties of the system. The downward shift in the rheological gelation temperature (ΔTgel = 0.3 °C) indicates that the liposomes may act as structural fillers or nucleating agents within the polymer network. The amphiphilic outer surfaces of the liposomes may interact with the block copolymers, thereby lowering the thermal energy barrier required for micellar close-packing and accelerating the sol-to-gel transition [36]. However, once the gel network fully matures at 32 °C, a distinct structural trade-off becomes apparent. In both the amplitude sweeps and 3ITT protocols, the GelLip exhibits a lower maximum storage modulus plateau (~191–220 Pa) than the Gel (~254–380 Pa). This suppression suggests that while the physical presence of the liposomes facilitates initial micellar ordering at lower temperatures, their large steric bulk may subtly disrupt the ultimate cross-linking density and spatial uniformity of the continuous polymer network at higher temperatures [22].

Interestingly, although the incorporation of nanoliposomes slightly reduced the storage modulus (G′) of GelLip compared with the blank hydrogel during oscillatory measurements, it resulted in higher zero-shear viscosity (η_0_) and yield stress (τ_0_) under rotational flow. This apparently divergent behavior may be related to the different deformation regimes probed by the two rheological approaches. Within the linear viscoelastic region, the relatively small oscillatory deformation primarily probes the elastic response of the intact Poloxamer 407 micellar network. The incorporation of dispersed nanocarriers may locally modify the organization and packing of the micellar network, resulting in a modest reduction in G′. Similar modifications of the rheological response have been reported for Poloxamer 407 hydrogels containing lipid-based nanocarriers and have been associated with interactions between the dispersed nanoparticles and the polymeric micellar structure [37,38]. In contrast, rotational flow progressively challenges the microstructure and probes the resistance of the formulation to structural rearrangement and macroscopic flow. The presence of the nanoliposome phase increases the microstructural complexity of the hydrogel and may introduce additional interfacial interactions with the Poloxamer matrix, thereby increasing the resistance to flow and contributing to the higher η_0_ and τ_0_ observed for GelLip [37,38]. Thus, the lower G′ and higher η_0_/τ_0_ should not be considered contradictory, as these parameters describe different aspects of the viscoelastic and flow behavior of the composite hydrogel.

Consistent with these complementary rheological characteristics, the mechanical properties of the GelLip retained favorable shear-dependent flow properties that may facilitate its administration through a nasal delivery device. For an in situ gelling nasal system to be clinically applicable, it must remain readily sprayable despite forming a highly viscous gel after administration. The rotational flow curves and 3ITT profiles at 32 °C provide evidence that both formulations meet this requirement through shear-thinning (pseudoplastic) and thixotropic behavior. Under increasing experimentally applied shear rates, the formulations exhibited a progressive reduction in apparent viscosity. Under these conditions, the entangled polymer micelles align parallel to the direction of flow, breaking weak secondary bonds and causing the complex viscosity to drop sharply, reaching a low baseline of ~220 mPa·s for the GelLip. This behavior is supported by the 3ITT high-shear interval, during which the GelLip experiences a deep structural breakdown, with its modulus dropping to 50 Pa. Interestingly, the GelLip undergoes a substantially greater structural breakdown under high shear than the Gel (G′ = 210 Pa). This suggests that the embedded liposomes may act as internal lubricating spheres that reduce friction within the sheared polymer network, which may facilitate atomization and promote uniform droplet distribution inside the nasal cavity [39].

Importantly, these favorable rheological properties at high shear may contribute to improved performance under post-deposition conditions, potentially helping to overcome the primary clinical limitation of conventional intranasal liquid formulations. Natural defense mechanisms, such as continuous mucus production and synchronous ciliary beating, drive high mucociliary clearance rates that routinely clear standard liquids within 15–20 min, severely limiting the time available for drug absorption. The GelLip developed in this study may help overcome this clearance barrier through its unique low-shear rheological properties at physiological temperatures. Immediately following contact with the warm nasal mucosa (32 °C), the formulation encounters a near-static environment characterized by very low shear rates (γ → 0). Under these conditions, the GelLip forms a highly structured matrix with a low-shear viscosity of 8000 mPa·s and a robust yield stress τ0 of 7.942 Pa, which is nearly eightfold higher than that of the Gel. This high-yield stress may act as a mechanical anchor, allowing the GelLip to resist gravity and the clearance forces of ciliary movement [22,40].

Furthermore, the 3ITT kinetics demonstrate that this structural recovery is rapid, with the elastic modulus increasing to 500 Pa within 50 s of shear cessation. This fast recovery may allow the formulation to form a stable, localized depot before it can be cleared through the nasopharynx. By prolonging nasal residence time and protecting encapsulated therapeutics from localized enzymatic degradation, this GelLip network may extend the window for transmembrane mucosal absorption. The prolonged retention of the liposomal nanocarriers at the olfactory epithelium and along trigeminal nerve pathways may facilitate continuous macromolecular translocation via intracellular axonal transport or extracellular bulk flow through perineural spaces. Consequently, the rheological properties of this composite platform may contribute to reduced peripheral clearance, prolonged mucosal contact, and ultimately, enhanced accumulation of therapeutic agents within targeted brain tissues [22,40].

The time-dependent, statistically significant decrease in the RFI signal in the nasal cavity observed for the non-gel formulations, but not for the gel formulations, may be attributed to the prolonged retention of the thermoresponsive gel within the nasal cavity. Due to the rheological properties of the thermoresponsive gel, clearance from the nasal mucosa may have been reduced. Additionally, its higher viscosity may have facilitated more efficient nasal deposition, reducing the amount of the administered formulation that was inhaled or swallowed. In contrast, the non-gel formulations were more fluid, potentially resulting in a greater proportion of the administered dose being inhaled rather than adhering to the nasal mucosal surface, consistent with the higher RFI signal detected in the lungs. The data in Figure 8D illustrate the difference between the non-gel and gel formulations.

These results indicate an improved nasal retention profile for the gel formulations, suggesting a longer residence time on the nasal mucosa compared with the non-gel formulations. Previous studies have reported that similar gel-based formulations can extend nasal retention time from minutes to hours [40,41].

The whole-brain RFI values demonstrate expected differences in the transport kinetics and potential pathways of the tested formulations. ICG as a free molecule is transported passively through paracellular spaces and via transcellular or intracellular pathways [42]. Encapsulation in liposomes favors cellular uptake through endocytic mechanisms [43]. In contrast, incorporation into a gel as a matrix promotes the local retention of ICG molecules on the nasal mucosa. This may explain why a difference was observed at 4 h between GelICG and the other tested formulations. The transport of LipICG and especially of GelLipICG may be influenced by the slower cellular uptake and transport kinetics associated with liposomal encapsulation. At later time points, GelLipICG reached the highest RFI values compared with the gel-only and liposome-only formulations and especially when ICG was applied as an aqueous solution. This may reflect the combined effect of the prolonged retention provided by the gel and the slower but sustained transport associated with the liposomes. At 24 h, the observed differences became statistically significant.

Several potential pathways may contribute to the transport of the intranasally applied formulations in this experiment from the olfactory mucosa toward the brain. First, an intracellular neuronal pathway may contribute, in which the formulation is internalized by olfactory receptor neurons through endocytosis and transported along axons toward the olfactory bulb. Second, transport may occur through extracellular or paracellular pathways, with the formulation moving through perineural spaces or across the epithelium toward the lamina propria. Third, transcellular epithelial transport may take place, involving passage through epithelial or supporting cells into the lamina propria, followed by further distribution through lymphatic drainage to cervical lymph nodes or entry into the systemic circulation through local blood vessels [5]. In the context of nose-to-brain delivery, the olfactory pathway, which is particularly relevant for delivery to the olfactory bulbs, frontal cortex, and cerebrum, and the trigeminal pathway, which may contribute to transport toward deeper brain regions such as brainstem, pons, cerebellum, and spinal cord, are considered the two main anatomical routes connecting the nasal cavity with the CNS [44].

The observed RFI signal from the OBs was several times higher than that from the brainstem for all time intervals for the GelICG, LipICG and GelLipICG formulations. Thus, we hypothesize that transport occurred predominantly through the olfactory pathway, which is consistent with published data. While the olfactory nerve endings are directly exposed to the nasal cavity, the trigeminal nerve branches are covered by the nasal mucosa. This anatomical characteristic may hinder access of the applied formulations, limiting their penetration and transport [44]. Additionally, the length and branching pattern of the trigeminal nerve may contribute to slower transport kinetics compared with the olfactory pathway [45]. Therefore, the olfactory mucosa is considered a major target for nose-to-brain delivery, which is consistent with our results. The measured RFI signal from the brainstem remained relatively stable over time compared with the fluctuations observed in the OBs for all tested formulations.

The gel formulations demonstrate significantly higher and faster transport through both pathways as the RFI signal in the OBs and brainstem is highest, especially for GelICG in the OBs at 4 h. Similarly, in the OBs, the GelLipICG group shows the highest transport but is delayed in time as its RFI signal is higher than in the rest of the experimental groups at 8 and 24 h. The delay is probably a result of the difference in size between a single ICG molecule and ICG-loaded liposomes. The same factor may also influence the preferred transport pathway [46,47].

The most convincing evidence that our thermoresponsive gel is suitable for enhanced, sustained intranasal delivery of ICG-(probably drug-) loaded nanoliposomes is the result at 24 h, where GelLipICG retained the highest RFI signal compared to all other formulations and is strongly supported by our previous study with this liposomal formulation, which demonstrated robust stability and efficient cellular uptake in vitro [48]. Most importantly, the signal is significantly higher than the liposome formulation without the gel, for both OBs and the rest of the brain tissue. However, additional microscopic histopathological safety profiling is required for subsequent translational milestones of this thermoresponsive platform. This is in line with the scientific literature showing that incorporation of the ICG into the gel or its encapsulation in liposomes prolongs the lifetime [49] and retention [50] of the dye in brain tissues.

As part of this experimental study, the RFI signals of selected internal organs were measured. The liver and gastrointestinal tract, which are associated with ICG elimination, showed the highest RFI values. At early time points, the non-gel formulations appeared to be cleared more rapidly than the gel formulations, suggesting altered systemic availability and clearance kinetics of ICG. At 24 h, the signals associated with all formulations were markedly reduced in the liver [51]. A similar pattern among the groups, although shifted in time, was observed in the gastrointestinal tract. The RFI signal intensity from the gastrointestinal tract was significantly higher than that from the liver for two possible reasons. First, it may reflect a combination of swallowed and biliary-excreted ICG. Second, ICG is rapidly cleared from the liver into the bile, whereas it may remain in the intestinal tract longer because of slower intestinal transit.

The RFI signal was also measured in the heart, lungs, and kidneys as organs potentially susceptible to side effects when using bioactive molecules incorporated into gels and/or liposomes. In a previous study, it was established that a protein corona is formed on the liposome surface, which affects the liposomal biodistribution, absorption, metabolism, and elimination. Specifically, among the proteins adsorbed onto the surface of the liposomes tested in this study, albumin was present at the highest proportion [43]. Our results suggest more rapid clearance and greater distribution to the heart and kidneys for LipICG compared with the other tested formulations. In the liver, organic anion influx transporters, ATP-dependent efflux transporters, and phospholipid/xenobiotic exporters participate in ICG clearance. Similar transport systems are expressed in the heart and kidneys, although these organs do not have the same excretory role as the liver [52,53,54,55,56,57,58]. The albumin–liposome complex may therefore interact with transport systems shared among the liver, heart, and kidneys, potentially contributing to the detectable signals observed for liposome-containing formulations.

## 4. Materials and Methods

### 4.1. Materials

Soybean lecithin (SL) was purchased from Vitalia, Skopje, Republic of North Macedonia. LIPOID PE 18:0/18:0-PEG 2000 (PEG) was obtained from Lipoid GmbH, Ludwigshafen, Germany. Cholesterol, HPLC-grade methanol, and HPLC-grade chloroform were purchased from Merck KGaA, Darmstadt, Germany. Poloxamer 407 and Poloxamer 188 were supplied by BASF, Ludwigshafen, Germany.

All reagents and materials were of the highest available purity, complied with the requirements of the relevant European Pharmacopoeia monographs, and were used as received without further purification or modification.

### 4.2. Preparation of ICG-Loaded Nanoliposomes

ICG-loaded nanoliposomes (LipICG) were prepared using a modified thin-film hydration method adapted from Shalabalija et al. (2021) [43]. Lecithin and cholesterol were used as the main lipid components in an approximate weight ratio of 9:1, while 50 mg of mPEG-2000 was incorporated to improve colloidal stability. The lipid components were dissolved in chloroform:methanol (4:1, *v*/*v*), and the organic solvents were removed under reduced pressure using a rotary evaporator (Rotavapor Buchi 215, Flawil, Switzerland) until a thin lipid film was formed.

The lipid film was hydrated with phosphate buffer pH 7.4 containing indocyanine green (ICG) at a concentration of 0.3 mg/mL. Hydration was facilitated by manual mixing, ultrasonication (ULTRASONS-H, J.P. Selecta), and vortexing (Tehtnica EV-102, Slovenia), each performed for 5 min. The resulting dispersion was homogenized using an Ultra-Turrax T25 homogenizer (IKA-Werke, Staufen im Breisgau, Germany) at 13,500 rpm for 3 min and stored at 2–8 °C for 24 h. After equilibration, the dispersion was additionally homogenized at 6500 rpm for 1 min. Non-encapsulated ICG was removed by three centrifugation washing cycles at 4000 rpm for 15 min at 25 °C. The purified nanoliposomal dispersion was filtered through a 0.45 µm membrane filter and stored at 2–8 °C, protected from light, until further use.

### 4.3. Preparation of Thermoresponsive Gel (Gel)

The thermoresponsive in situ gel or blank hydrogel matrix (Gel) was prepared by gradual dispersion of the polymeric components in purified water under continuous magnetic stirring. Poloxamer 407 and Poloxamer 188 were used as thermoresponsive polymers in an optimized weight ratio of 3:1 (*w*/*w*), corresponding to final concentrations of 22.5% and 7.5% (*w*/*w*), respectively, resulting in a total Poloxamer concentration of 30.0% (*w*/*w*) in the 20 g formulation. Low molecular weight hyaluronic acid and Carbomer 940 were subsequently incorporated as mucoadhesive components at 0.05% (*w*/*w*) each (10 mg each per 20 g of formulation). The formulation was adjusted to the final mass with purified water and stirred overnight to ensure complete polymer hydration and formation of a homogeneous gel base.

The final pH of the nasal gel was adjusted to 5.5 using sodium hydroxide (NaOH). The pH was selected within the commonly reported range for intranasal formulations (4.5–6.5) to minimize potential irritation of the nasal mucosa. The prepared gel was stored at 2–8 °C until further use. Before incorporation of nanoliposomes, the formulation was visually inspected for homogeneity and absence of visible aggregates. The gel remained liquid at room temperature and was designed to undergo a sol-to-gel transition at physiological nasal temperature.

### 4.4. Incorporation of Nanoliposomes into the Thermoresponsive Gel (GelLip)

Blank nanoliposomes or ICG-loaded nanoliposomes were incorporated into the thermoresponsive gel (GelLip or GelLipICG, respectively) under mild mixing conditions to preserve nanoliposome integrity. The thermoresponsive gel and nanoliposomal dispersion were combined in a mass ratio of 3:1 (gel:nanoliposomal dispersion). Briefly, the Gel was equilibrated to room temperature to maintain its liquid state, after which the nanoliposomal dispersion was gradually added under gentle stirring until a homogeneous nanoliposome-loaded thermoresponsive formulation was obtained. The final formulation was stored at 2–8 °C and protected from light until use. Before in vitro experiments or intranasal administration, the formulation was equilibrated to room temperature to ensure appropriate flow properties for application. Upon exposure to physiological nasal temperature, the system underwent sol-to-gel transition, forming a mucoadhesive gel matrix with uniformly dispersed nanoliposomes.

### 4.5. Characterization of ICG-Loaded Nanoliposomes

#### 4.5.1. Particle Size and Size Distribution

The particle size and size distribution of the ICG-loaded nanoliposomes (LipICG) were determined by laser diffraction using a Mastersizer 2000 equipped with a Hydro 2000S dispersion unit (Malvern Instruments Ltd., Malvern, UK). An aliquot of each nanoliposomal dispersion was added to the measurement cell containing distilled water as the dispersing medium until an appropriate obscuration level was achieved. Measurements were performed under continuous stirring at 1750 rpm to ensure uniform dispersion of the sample during analysis.

The mean particle size was expressed as D50, while the width of the particle size distribution was expressed as SPAN. To ensure reproducibility, at least three independently prepared batches were analyzed, with a minimum of three measurements performed for each batch. Each measurement represented the average of ten consecutive runs.

Complementary particle size, polydispersity and surface charge measurements were performed by dynamic light scattering (DLS) using a Zetasizer Nano-ZS (Malvern Instruments Ltd., Malvern, UK). Prior to analysis, the samples were diluted 20-fold with 10 mM phosphate buffer. Measurements were performed at 25 °C after a 120 s equilibration period, using a backscatter detection angle of 173°. Particle size distribution was expressed as the polydispersity index (PDI), while surface charge was expressed as zeta potential (mV). At least three independently prepared batches were analyzed, with each sample measured in triplicate.

#### 4.5.2. Encapsulation Efficiency of ICG Loading

The encapsulation efficiency of ICG was determined indirectly by quantifying the non-encapsulated dye fraction using a validated spectrophotometric method. The spectrophotometric procedure was adapted from previously reported UV–Vis methods for ICG quantification and its suitability was verified under the present experimental conditions. Selectivity was confirmed using buffer and blank nanoliposomes without ICG. Briefly, the nanoliposomal dispersion was subjected to centrifugal ultrafiltration using membrane units with a molecular weight cut-off of 100,000 Da. The separated filtrate, containing non-encapsulated ICG, was collected after centrifugation, and the membrane units were rinsed with buffer to ensure complete recovery of the free dye fraction. This fit-for-purpose procedure provided the necessary selectivity, sensitivity, and precision for characterising the free dye fraction in the ultrafiltrate. In line with the scope of this formulation and biodistribution study, the method was utilized as a verified screening tool rather than a fully validated analytical protocol for complex matrix extraction.

The concentration of ICG in the collected filtrate was determined spectrophotometrically at 805 nm. The amount of non-encapsulated ICG was then used to calculate encapsulation efficiency relative to the total amount of ICG initially added during formulation preparation. ICG loading was calculated based on the amount of encapsulated ICG relative to the final nanoliposomal formulation content. ICG loading was calculated as the amount of encapsulated ICG relative to the total mass of lipid components used for nanoliposome preparation (lecithin, cholesterol, and mPEG-2000) and expressed as percentage weight/weight (%*w*/*w*).

### 4.6. Rheological Characterization

Comprehensive rheological profiling of the thermoresponsive gel (Gel) and the thermoresponsive gel loaded with nanoliposomes (GelLip) was executed using a controlled-stress rotational and oscillatory rheometer (Anton Paar MCR 92) configured with an integrated Peltier temperature control system. All oscillatory and rotational measurements were completed using an optimized plate-cone geometry system. Before data acquisition, samples were carefully loaded onto the lower plate and allowed a 2-min equilibration period to achieve structural equilibrium and thermal normalization.

#### 4.6.1. Linear Viscoelastic Region (LVR) and Amplitude Sweeps

To isolate the non-destructive linear viscoelastic region (LVR) and evaluate critical structural sensitivity thresholds, oscillatory amplitude sweeps were carried out at both ambient temperature (25 °C) and simulated physiological nasal temperature (32 °C). The tests were conducted at a fixed angular frequency (ω) over a logarithmically increasing shear strain (γ) sweep ramping from 0.01% to 100%. Viscoelastic parameter profiles—specifically storage modulus (G′), loss modulus (G″), complex viscosity (η*) and the structural loss factor (tanδ = G″/G′) were plotted as a function of shear strain. The boundary limit of structural stability, designated as the critical shear strain threshold (γc), was identified at the point where G′ deviated by more than 10% from its initial horizontal LVR plateau.

#### 4.6.2. Thermal Phase Transition Dynamics (Temperature Sweeps)

The sol-to-gel transition kinetics and temperature-dependent self-assembly profiles of the polymeric hydrogel matrices were investigated via continuous oscillatory temperature ramps. The experimental temperature range scaled progressively from ambient storage conditions (~20 °C) up to hyperthermic body limits (~40 °C) at a uniform thermal heating rate. The testing parameters were maintained strictly within the previously established LVR strain boundaries using a constant angular frequency. The rheological gelation temperature (Tgel) was mathematically extracted as the exact thermodynamic crossover point where the viscoelastic parameters converged and inverted, satisfying the condition:

*tanδ* = *G*″/*G*′ = 1.0

The ultimate network strength parameters were evaluated across the elevated physiological plateau, tracking terminal storage modulus limits up to approximately 39.5 °C.

#### 4.6.3. Steady-State Rotational Flow Curve Analysis

Steady-state rotational rheology profiles were measured at physiological nasal temperature (32 °C) to characterize the flow behavior and shear-dependent viscosity of the formulations. The experimental run subjected the formulations to a continuous, logarithmic shear-rate sweep (γ) from 1 to 1000 s^−1^. Apparent dynamic viscosity (η) was continuously monitored as a function of shear rate to characterize the Newtonian or non-Newtonian flow behavior, including pseudoplastic (shear-thinning) behavior. To quantify the flow parameters, the experimental shear stress (τ) versus shear rate (γ) curves were fitted to the Herschel–Bulkley model.

#### 4.6.4. Three-Step Interval Thixotropy Testing (3ITT)

To evaluate the microstructural breakdown under mechanical stress and subsequent structural recovery kinetics upon post-administration deposition, a 3-Step Interval Thixotropy Test (3ITT) was deployed. The test sequence was evaluated at both ambient conditions (25 °C) and nasal physiological temperature (32 °C), consisting of three discrete, sequential operational phases: Interval 1 (Low-Shear Baseline Evaluation)—the formulation was maintained under a non-destructive low-shear oscillatory strain profile for a set duration to document initial resting viscoelastic parameters (G′, G″); Interval 2 (High-Shear Actuation Simulation)—the structure was subjected to a high-rate destructive shear deformation step; Interval 3 (Kinetic Structural Rebuilding Profile)—the destructive shear field was instantaneously halted, returning the system to original baseline low-shear conditions. The time-dependent rebuilding kinetics, tracking structural crossover events (G′ ≫ G″) and storage modulus recovery magnitudes, were monitored continuously over a 200-s resting phase.

### 4.7. Ex Vivo Biodistribution Study

All experimental procedures were conducted in accordance with the Guiding Principles for the Care and Use of Laboratory Animals and were approved by the Bulgarian Center for Bioethics and the Ethics Committee for Animal Experiments of Sofia University. The study also complied with the International Guiding Principles for Biomedical Research Involving Animals issued by the Council for International Organizations of Medical Sciences. We have ethical approval from the Bulgarian Food Safety Agency, Ministry of Agriculture, Food and Forestry (No. 381, 12 March 2024) and from the local Ethics Committee of the Faculty of Biology, Sofia University “St. Kliment Ohridski” (Protocol No. 3, 12 February 2024).

The ex vivo biodistribution study was performed to evaluate the time-dependent brain, nasal cavity, and internal organ distribution of ICG after intranasal administration of ICG-loaded nanoliposomes either as a liquid dispersion or incorporated into the thermoresponsive gel formulation. Free ICG was included as a control and monitored under the same experimental conditions and at the same time points as the ICG-loaded formulation to account for possible time-dependent changes in fluorescence arising from dye photodegradation or concentration-dependent fluorescence effects.

Three-month-old Wistar rats housed in the Faculty of Biology vivarium under a 12-h light/12-h dark cycle, with free access to food and water, were used in the experiment. Eighty-four animals were randomly assigned to four groups before the start of the experiment: aqueous solution (aqICG) weighed 295.61 ± 18.13 g (n = 21), ICG incorporated into a thermoresponsive gel (GelICG) weighed 244.61 ± 13.38 g (n = 21), ICG encapsulated in nanoliposomes (LipICG) weighed 305.2 ± 22.86 g (n = 21), and ICG-loaded nanoliposomes incorporated into a thermoresponsive gel (GelLipICG) weighed 325.13 ± 19.38 g (n = 21). Animals of both sexes were used in equal numbers in each group. To minimize transfer-induced stress before the procedures, the animals were moved to the adjacent experimental room within their original home cages and existing social groups.

Each animal received a single intranasal dose of 40 µL, administered into one nostril, containing 3 µg of ICG per animal, either in gel or non-gel formulation. No macroscopic bleeding, irritation, redness, swelling, breathing difficulties, or other signs of effects on the nasal mucosa were observed with all administered forms. The animals were under light anaesthesia during administration; they woke up within 5–10 min and remained active throughout the entire testing period. The animals were further divided into subgroups according to the time point of tissue collection: 4 h, 8 h, and 24 h after intranasal administration. The final sample size was set at n = 6 successfully treated animals per subgroup (total N = 72 analyzed animals), determined via power analysis to detect a large effect size (f = 0.40) at α = 0.05 and 80% power. A pre-defined exclusion criterion was established: animals with technical complications during formulation delivery (residual volume in cannula, exhaled droplets, etc.) were excluded immediately. In accordance with ethical guidelines, all animals in which the application was compromised were not sacrificed and were returned to the facility for educational purposes. Only groups with exactly n = 6 successfully treated animals progressed to the scheduled time-point sacrifices and further analysis.

Before intranasal administration, the animals were anesthetized using an inhalation anesthetic consisting of isoflurane (Baxter, Sofia, Bulgaria) and oxygen. Isoflurane was applied at 4% for induction, followed by 1.5% for maintenance for 5 min. The animals were subsequently intraperitoneally injected with Nalgosed at 2 mg/kg (Bioveta, a.s. (C), Ivanovice na Hané, Czech Republic) and Sedin at 3.75 mg/kg (Vetpharma, Barcelona, Spain). This follows the anesthesia approach used in the previous intranasal ICG/PLGA biodistribution study [27,59].

All formulations were administered via a venous cannula (24 G × 34″; ⌀ 0.7 × 19 mm; KD-FIX, Berlin, Germany) to the upper third of the nasal cavity to maximize deposition in the region adjacent to the olfactory mucosa. This administration site was selected because the olfactory mucosa is directly connected with the olfactory bulbs through olfactory receptor neurons, while trigeminal nerve endings in the nasal mucosa provide an additional route toward the brainstem. Therefore, the olfactory bulbs and brainstem were selected as key regions for evaluating the involvement of the principal nose-to-brain delivery pathways.

The gel formulation was applied in its liquid state at room temperature. Upon contact with the nasal mucosa, where the physiological temperature is approximately 32 °C, the formulation was expected to undergo a sol-to-gel phase transition.

At 4 h, 8 h, and 24 h after administration, the animals were anesthetized via inhalation according to the protocol described above, after which they were decapitated. The nasal cavities, brains, and internal organs (liver, digestive system, heart, lungs, and kidneys) were carefully isolated to assess peripheral biodistribution and systemic elimination-related fluorescence. The temporal and nasal bones were removed, and the brain was gently excised to preserve the anatomical structures required for regional fluorescence analysis.

The brain was analyzed both as a whole organ and by selected regions associated with the main nose-to-brain transport pathways. The olfactory bulbs were analyzed as the primary region associated with olfactory pathway transport, while the brainstem was analyzed as a region associated with trigeminal pathway contribution. Fluorescence detected in other brain areas was used to evaluate the broader intracerebral distribution of the ICG-associated signal beyond the initial entry-related regions.

Near-infrared emission. NIR emission was measured using the Pearl Trilogy Small Animal Imaging System (LI-COR Biotechnology GmbH, Bad Homburg, Germany). ICG exhibits Stokes-shifted luminescence, with excitation in the 750–800 nm range and an emission peak at approximately 830 nm. In the present study, the acquisition parameters were strictly standardized utilizing the instrument’s dedicated 800 nm channel (optimized for ICG detection with a solid-state laser excitation source at 785 nm and an emission detection bandpass filter centered at 820 nm). To eliminate operator-dependent bias and ensure absolute quantification objectivity, the exposure settings were kept uniform across all images to maintain consistent signal capture across samples. Fluorescence intensity was quantified using Image Studio v6.0 software, Pearl Trilogy system. Penetration and distribution in the brain and selected internal organs were assessed by measuring NIR fluorescence intensity relative to the background. The signal was expressed as the difference between the fluorescence intensity of the selected region of interest and the corresponding background area. RFI values for distribution were normalized to the equal surface of the corresponding area according to the recommendation of the Image Studio Software. Regions of interest were defined for the whole brain, olfactory bulbs, brainstem, nasal cavities, and organs (equal in shape and size for each area). Corresponding background fluorescence was subtracted by the software from each measurement. This standardized protocol ensured that the calculated RFI values remained completely independent of the operator, providing an inherently unbiased automated evaluation.

To estimate ICG distribution in brain regions outside the main entry-associated areas (residual signal), the normalized fluorescence signal from the olfactory bulbs and brainstem was subtracted from the normalized whole-brain signal, divided by the residual area (the area of the whole brain minus the sum of the areas of the olfactory bulbs and brainstem).

$$\mathrm{RFI}=\frac{{\mathrm{Signal}}_{\mathrm{Whole}\;\mathrm{Brain}}-\left({\mathrm{Signal}}_{\mathrm{Olfactory}\;\mathrm{Bulbs}}+{\mathrm{Signal}}_{\mathrm{Brainstem}}\right)}{{\mathrm{Area}}_{\mathrm{Whole}\;\mathrm{Brain}}-\left({\mathrm{Area}}_{\mathrm{Olfactory}\;\mathrm{Bulbs}}+{\mathrm{Area}}_{\mathrm{Brainstem}}\right)}$$

Statistical analysis. Statistical analysis was performed using R statistical programming software v.4.4.0, RStudio version 2026.07.0+139 (“Pacific Dogwood” Release 9 July 2026). All data are presented as mean ± SEM. Differences between groups were assessed using Two-Way ANOVA, followed by Bonferroni’s post-hoc test for multiple comparisons. Differences were considered statistically significant at adjusted *p* < 0.05.

## 5. Conclusions

Overall, the developed thermoresponsive gel loaded with nanoliposomes demonstrated suitable physicochemical, rheological, and in vivo performance for intranasal nose-to-brain delivery. ICG-loaded nanoliposomes were successfully prepared with nanoscale particle size, narrow unimodal distribution, and high encapsulation efficiency of ICG, confirming their suitability as fluorescent nanocarriers for biodistribution studies. Incorporation of the nanoliposomes into the thermoresponsive gel preserved formulation homogeneity while providing temperature-dependent sol-to-gel transition, shear-thinning behavior, and rapid structural recovery, supporting its applicability for intranasal administration.

Our data demonstrate that intranasally administered ICG-loaded nanoliposomes slowly but persistently cross the rat nasal mucosal barrier and accumulate in the rat brain. Initially, they reach the olfactory bulbs and brainstem before subsequently distributing to other brain regions, consistent with observations reported for other nose-to-brain delivery platforms. Intranasal administration of ICG-loaded nanoliposomes using a thermoresponsive gel delayed their distribution within the rat brain but promoted sustained and more efficient nose-to-brain transport from the nasal mucosa; at 24 h, the RFI signal from the whole brain and residual brain tissues was strongest and significantly higher compared to the rest of the experimental groups studied. The sustained presence of the ICG signal in the brain over 24 h further supports the potential of this formulation to facilitate prolonged brain exposure to the agent, thereby maintaining drug exposure within the brain over an extended period. The good stability of nanoliposomes and their high accumulation in the gastrointestinal tract provide evidence that the observed NIR signal is mainly due to the penetration of ICG in nanoliposome form.

Most importantly, the gel-incorporated nanoliposomes produced a distinct and more sustained brain biodistribution profile compared with liquid nanoliposomes. Although the liquid formulation showed faster early transport, the gel formulation enabled prolonged and progressive brain fluorescence, with more pronounced effects in the measurements. These findings suggest that the thermoresponsive gel not only improves nasal residence but may also modulate nanoliposome-mediated nose-to-brain transport and spatial brain distribution.

Therefore, the combination of nanoliposomes and a thermoresponsive gel represents a promising delivery platform for the brain-directed delivery of non-permeable molecules and supramolecular structures. While ICG was utilized here as a model tracking agent, this carrier system demonstrates features suitable for the sustained delivery of therapeutics which may be beneficial for future applications requiring prolonged drug retention within the central nervous system.

## 6. Patents

An international patent application resulting from the work reported in this manuscript was filed under the Patent Cooperation Treaty (PCT) as International Application No. PCT/IB2026/058485 on 10 August 2026.

## Data Availability

All data generated or analyzed during this study are included in this published article.

## Abbreviations

The following abbreviations are used in this manuscript:

3ITT	3-Step Interval Thixotropy Test
aqICG	ICG in Aqueous Solution
CNS	Central Nervous System
Gel	Thermoresponsive gel
GelICG	ICG into a Thermoresponsive Gel
GelLip	Nanoliposomes loaded into a Thermoresponsive Gel
GelLipICG	ICG-loaded Nanoliposomes into a Thermoresponsive Gel
ICG	Indocyanine Green
LipICG	ICG in Nanoliposomes
LVR	Linear Viscoelastic Region
NIR	Near Infrared
OB	Olfactory Bulbs
PEO	Poly(Ethylene Oxide)
PLGA	Poly(D, L-Lactide-co-Glycolide)
PPO	Poly(Propylene Oxide)
RFI	Relative Fluorescence Intensity
SEM	Standard Error of the Mean
Tgel	Rheological Gelation Temperature
